# Metabolomic Analysis of Serum and Tear Samples from Patients with Obesity and Type 2 Diabetes Mellitus

**DOI:** 10.3390/ijms23094534

**Published:** 2022-04-20

**Authors:** Erdenetsetseg Nokhoijav, Andrea Guba, Ajneesh Kumar, Balázs Kunkli, Gergő Kalló, Miklós Káplár, Sándor Somodi, Ildikó Garai, Adrienne Csutak, Noémi Tóth, Miklós Emri, József Tőzsér, Éva Csősz

**Affiliations:** 1 Proteomics Core Facility, Department of Biochemistry and Molecular Biology, Faculty of Medicine, University of Debrecen, 4032 Debrecen, Hungary; erdenetsetseg.n@med.unideb.hu (E.N.); guba.andrea@med.unideb.hu (A.G.); kumar.ajneesh@med.unideb.hu (A.K.); kunkli.balazs@med.unideb.hu (B.K.); kallo.gergo@med.unideb.hu (G.K.); tozser@med.unideb.hu (J.T.); 2 Doctoral School of Molecular Cellular and Immune Biology, University of Debrecen, 4032 Debrecen, Hungary; 3 Department of Internal Medicine, Faculty of Medicine, University of Debrecen, 4032 Debrecen, Hungary; kaplar.miklos@med.unideb.hu (M.K.); somodi@belklinika.com (S.S.); 4 Department of Emergency Medicine, Faculty of Medicine, University of Debrecen, 4032 Debrecen, Hungary; 5 Department of Medical Imaging, Faculty of Medicine, University of Debrecen, 4032 Debrecen, Hungary; garai@belklinika.com (I.G.); emri.miklos@med.unideb.hu (M.E.); 6 Scanomed Ltd., Nuclear Medicine Centers, 4032 Debrecen, Hungary; 7 Department of Ophthalmology, Medical School, University of Pécs, 7632 Pécs, Hungary; acsutak@med.unideb.hu (A.C.); tothnoemi111@gmail.com (N.T.); 8 Department of Ophthalmology, Faculty of Medicine, University of Debrecen, 4032 Debrecen, Hungary

**Keywords:** amino acid, biogenic amine, obesity, type 2 diabetes, network analysis

## Abstract

Metabolomics strategies are widely used to examine obesity and type 2 diabetes (T2D). Patients with obesity (*n* = 31) or T2D (*n* = 26) and sex- and age-matched controls (*n* = 28) were recruited, and serum and tear samples were collected. The concentration of 23 amino acids and 10 biogenic amines in serum and tear samples was analyzed. Statistical analysis and Pearson correlation analysis along with network analysis were carried out. Compared to controls, changes in the level of 6 analytes in the obese group and of 10 analytes in the T2D group were statistically significant. For obesity, the energy generation, while for T2D, the involvement of NO synthesis and its relation to insulin signaling and inflammation, were characteristic. We found that BCAA and glutamine metabolism, urea cycle, and beta-oxidation make up crucial parts of the metabolic changes in T2D. According to our data, the retromer-mediated retrograde transport, the ethanolamine metabolism, and, consequently, the endocannabinoid signaling and phospholipid metabolism were characteristic of both conditions and can be relevant pathways to understanding and treating insulin resistance. By providing potential therapeutic targets and new starting points for mechanistic studies, our results emphasize the importance of complex data analysis procedures to better understand the pathomechanism of obesity and diabetes.

## 1. Introduction

One of the most widespread metabolic disorders worldwide is diabetes, and more than 90% of people with diabetes are diagnosed with type 2 diabetes (T2D). According to the International Diabetes Federation, 537 million individuals worldwide suffer from diabetes now, with that figure anticipated to rise to 643 million in 10 years and 783 million by 2045, according to their prediction in 2021 [1]. 

Extensive research has proven that obesity is the main leading risk factor for the development of T2D [2,3,4,5]. The probability of developing T2D was seven times higher in individuals with obesity and three times higher in overweight subjects [6,7]. Most people with T2D are overweight and obese with central visceral adiposity [8], indicating an important role for the adipose tissue and obesity in the development of T2D.

Regarding the pathophysiology of obesity-induced T2D, two main factors have been implicated: insulin resistance and beta-cell dysfunction. Insulin resistance is defined as an impaired response of the body to insulin action, despite insulin being at a higher or normal level [9,10]. This means that the blood plasma insulin level of individuals with T2D can be in the normal range, but the insulin is not able to stimulate glucose utilization by the cells, leading to hyperglycemia [11,12]. 

Being obese for a prolonged period leads to a permanently elevated glucose concentration in the bloodstream and, subsequently, to increased insulin production in the beta-cells as a compensatory mechanism for a hyperglycemic state. Over time, beta-cells are retarded and insulin secretion decreases [3,7]. 

T2D is generally characterized by hyperglycemia. It can be diagnosed by determining glucose levels in the bloodstream [13,14], but monitoring blood glucose levels alone may not be sufficient to better characterize the complex biochemical picture. 

For many years, extensive studies on the metabolism of carbohydrates, proteins, and lipids [15,16] have achieved great success in elucidating the role of these molecules involved in the pathophysiology of obesity and T2D. Most recently, metabolomics studies have been conducted to explore the importance of amino acids, which provide us with very specific information about many cellular functions such as carbohydrate and lipid metabolism, and protein synthesis involved in the pathophysiology of T2D [13,17,18,19,20]. 

In recent decades, advanced technologies, such as mass spectrometry (MS) coupled with ultra- or high-performance liquid chromatography (UPLC or HPLC), gas chromatography, and nuclear magnetic resonance spectroscopy were intensively applied in many fields of research areas, including the clinical diagnosis of diseases, biomedical studies, pharmacology, and food science [21,22]. Thus, the application of technological advances in the study of T2D and obesity allows us to determine the trace amount of metabolites that might expand our understanding of the disease [23].

Due to the demand of future prognoses, prevention of complications, and finding affordable treatment options, researchers have focused on protein and metabolite profiling in different types of body fluids, including serum, plasma, saliva, tear, or urine samples from patients with obesity or T2D [13,15,24,25,26,27,28].

Amino acids such as isoleucine, leucine, valine, and glycine were associated with the risk of development of T2D [29] and obesity [30], and correlations between the level of some amino acids with insulin resistance or glucose level were demonstrated [31]. Biogenic amines generated from amino acids by decarboxylation were also studied. The serum level of putrescine was shown to be elevated in T2D and correlated with the level of glycated hemoglobin (HbA1C) [32]. In a cell culture study, methylamine, a biogenic amine found in food, was found to activate glucose uptake in adipocytes [33]. Other biogenic amines such as spermidine, kynurenine, and creatinine were associated with the transition from gestational diabetes mellitus to T2D [18], and the level of tyramine in the urine was found to be decreased in patients with metabolic syndrome compared to controls [34]. 

It is well known that the chronic metabolic changes characteristic of T2D can lead to numerous complications, which are generally grouped into macrovascular and microvascular diseases [35]. One of the most common eye-related microvascular complications of T2D is diabetic retinopathy (DR) which, if untreated, may lead to blindness [36]. Potential biomarkers such as nerve growth factor, apolipoprotein (Apo) A1, lipocalin 1, lactotransferrin, lacritin, lysozyme C, lipophilin A, immunoglobulin lambda chain, HSP27, and β2-microglobulin in tears were identified as being specific to DR, associated either negatively or positively with the condition [27,37,38,39]. 

Tear fluid may be an ideal source for biomarker discovery concerning DR due to its unique composition, easy collection, proximity to the disease location, and minimal cell contamination [40,41]. Since the sample collection method is non-invasive for patients and easy for technicians [42], tears can be an attractive sample choice for metabolite analyses in diabetic patients. 

As amino acids and biogenic amines have an important role in metabolic functions, in addition to being deeply involved in the pathophysiology of obesity and T2D, we aimed at profiling amino acids and biogenic amines in serum and tear samples of patients with T2D, patients with obesity, and of a matched control group. We applied a complex data analysis workflow and a network model to examine metabolic networks. This type of analysis can provide a new perspective in the assessment and interpretation of metabolomics data and the understanding of the pathophysiological mechanism driving obesity and T2D.

## 2. Results and Discussion

T2D and obesity are pathological conditions affecting the life quality of millions of people worldwide [43,44]. Metabolomics is a widely used analytical approach to examine the metabolic alterations reflected at the level of body fluids such as serum or tears [21,40,45,46,47].

### 2.1. Serum Metabolomics in Obesity and T2D

The examination of metabolites found in serum is a routinely used method in laboratory diagnostics. The examination of the lipid panel and determination of the level of some proteins and small molecules are part of the routine diagnostics used to monitor the well-being of patients or to detect the asymptomatic progression of diabetes or obesity [16,23,34]. In our study, the serum collected from the recruited donors was subjected to well-established clinical laboratory examinations. The levels of blood glucose, HbA1C, triglyceride, cholesterol, high-density lipoprotein (HDL), low-density lipoprotein (LDL), ApoA1, ApoB100, insulin, C peptide, C-reactive protein (CRP), and fibrinogen was measured, and the glomerular filtration rate (GFR) and the homeostatic model assessment of insulin resistance (HOMA-IR) were checked. The circumference of the abdomen, waist, and neck was measured in patients, and the body-mass index (BMI) and the waist–to–hip ratio (WHR) were calculated. 

Besides the clinical laboratory tests, the levels of 23 amino acids and 10 biogenic amines were analyzed. The results of the clinical and laboratory analyses along with the demographic data are presented in Table S1.

#### 2.1.1. Examination of the Concentration of Amino Acids

In the last decade, several studies were carried out examining the metabolites characteristic of obesity and/or T2D to find predictors of T2D and further complications [13,47,48,49,50]. Amino acids were extensively studied and, in all cases, the involvement of branched-chain amino acids (BCAA) and glycine were concluded. The levels of leucine, isoleucine, and, in some cases, valine increased, whereas the level of glycine decreased in T2D compared to controls [51,52,53,54,55]. 

**Figure 1:**
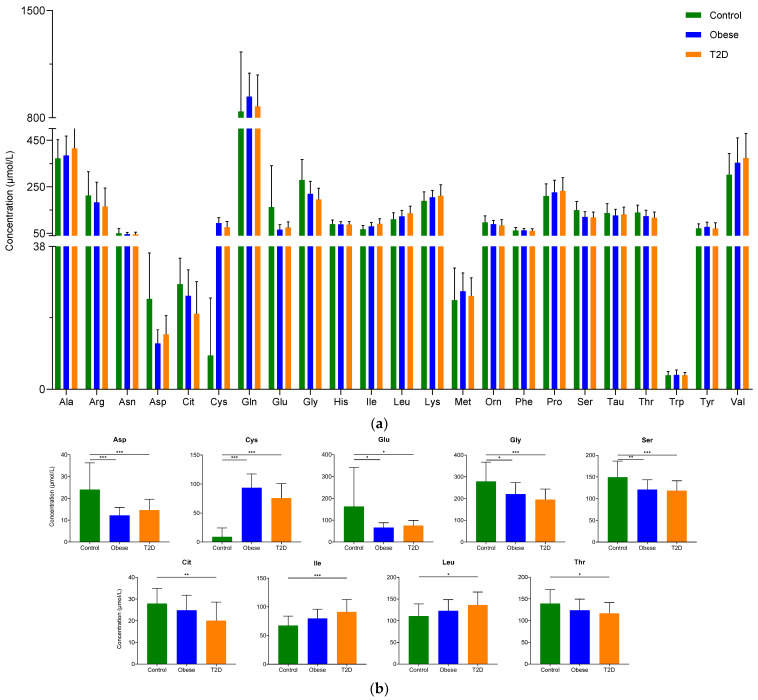
The serum concentration of examined amino acids: (**a**) The concentration of amino acids in serum. The y-axis shows the concentration in µmol/L of amino acids shown on the x-axis; (**b**) The concentration of amino acids showing statistically significant changes between the groups. The y-axis represents the concentration of individual amino acids, and the x-axis indicates the examined patient groups. * *p*-value ≤ 0.05; ** *p*-value ≤ 0.01; *** *p*-value ≤ 0.001. The green color refers to the control group, the blue color represents the obese group, and orange indicates the T2D group.

In our study, we could detect and quantify all the analyzed 23 amino acids in sera from donors belonging to control, obese, and T2D groups (Figure 1a, Table S1). Using statistical analysis, the amino acids that showed a statistically significant change between the groups were determined (Figure 1b). The levels of nine amino acids changed in a statistically significant manner between the control and disease groups. We observed an increase in the level of cysteine and a reduction in the levels of aspartate, glutamate, glycine, and serine in the obese group compared to the control. Regarding the difference between the control and T2D groups, we noted elevated levels in the cases of cysteine, isoleucine, and leucine, while the concentrations of aspartate, citrulline, and glutamate, glycine, serine, and threonine dropped in the T2D group compared to the control group. 

Our findings are in agreement with data published in the scientific literature; we observed significantly higher levels of isoleucine and leucine and lower concentrations of glycine in patients with T2D compared to controls. 

To collect all the available information, we searched the scientific literature and collected the studies showing a statistically significant change in the concentration of amino acids between the control and either obese or T2D groups (Table S2 and references shown therein). According to the table, the increase in alanine, glutamate, isoleucine, phenylalanine, tyrosine, and valine and the decrease in glutamine and serine in T2D compared to the control was statistically significant in all found studies, while in the cases of arginine, glutamine, glycine, and leucine most of the studies indicate changes in one direction, but there is at least one study showing changes in the opposite direction. Two studies indicate increased methionine levels, and one shows elevated proline levels in T2D, but regarding the other amino acids, approximately the same number of studies show an increase or decrease in T2D compared to controls (Table S2 and references listed there). We found similar results in studies examining the amino acid levels in obesity.

#### 2.1.2. Examination of the Concentration of Biogenic Amines

In contrast to amino acids, little data is available on the effect of biogenic amines in diabetes or their potential as biomarkers in obesity or T2D. Studies published in the scientific literature show a significant increase in serum putrescine concentration in T2D [32], elevated concentrations of serotonin in the urine of patients with T2D [56], and decreased concentration of tyramine in the urine of patients with metabolic syndrome compared to controls [34]. 

**Figure 2:**
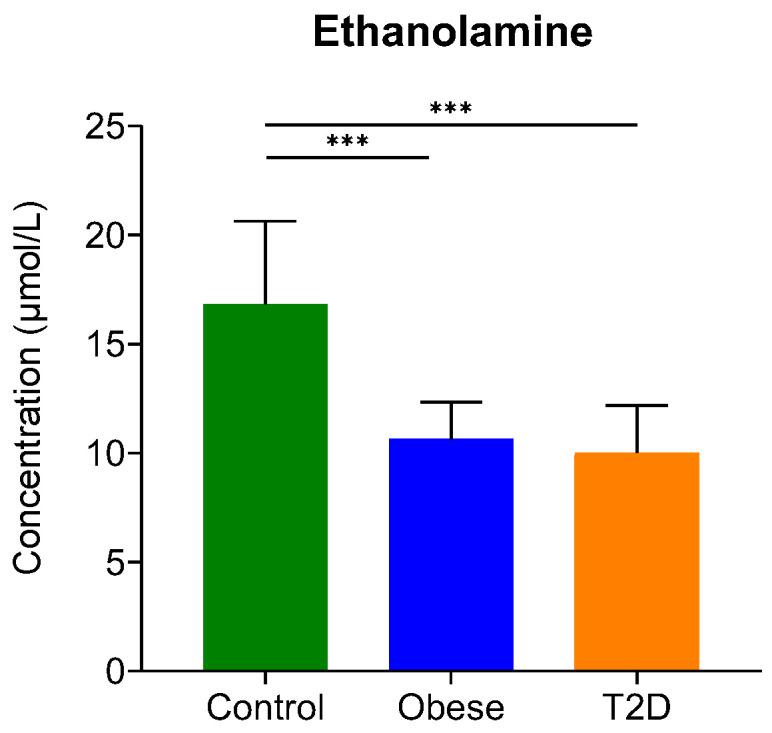
The serum concentration of ethanolamine. The y-axis represents the concentration in µmol/L of biogenic amine on the x-axis. ***, *p*-value ≤ 0.001. The green color refers to the control group, the blue color represents the obese group, and the orange indicates the T2D group.

In our study, the level of 10 biogenic amines was examined with regard to T2D and obesity (Table S1). Ethylamine, putrescine, and serotonin could be detected, but their level was very low, lower than the limit of quantification, so we could only detect their presence in serum without being able to quantify them (Table S1). Ethanolamine and, in some samples, methylamine were present in higher amounts, so their quantification was possible in the serum of donors (Figure 2). In the case of ethanolamine, we observed statistically significant differences: its level lowered in both disease groups compared to control. The decrease in ethanolamine levels was shown to be a cerebrospinal fluid biomarker for major depressive disorder [57], but no statistically significant change in its level between T2D and control groups could be detected [58] so far. However, if we consider that ethanolamine at the same time is a precursor and a metabolite of anandamide, our findings are not surprising. By its relation to the endocannabinoid signaling implicated in the pathomechanism of diabetes [59], serum ethanolamine can be considered a biomarker for insulin resistance, which is present both in obesity and T2D.

The quantification of methylamine was possible only in six samples (Table S1), so we omitted methylamine from further analyses. Other biogenic amines including histamine, cadaverine, tyramine, and phenethylamine were not detected in the serum.

#### 2.1.3. Correlation Analysis

Having information on the features changing together in a system might help to better understand the complex metabolic landscape governing the pathology of obesity and diabetes. To obtain more insights into these subtle associations, a correlation analysis was carried out. Using Pearson correlation analysis, we examined which of the previously recorded clinical parameters correlate with the concentration of the examined amino acids and biogenic amines. As seen in our data (Table 1 and Table S3), a negative correlation of both serum insulin and C peptide levels with glycine and serine concentrations could be confirmed. Similarly, there was a negative correlation between HOMA-IR and glycine levels. These data agreed with previous findings, indicating decreased glycine levels as characteristic of insulin resistance [60]. The alterations at multiple levels of glycine and serine metabolism were shown to be important in obesity and T2D [61]. Glycine and serine can be interconverted into each other via the serine-hydroxymethyl transferase (SHMT) enzymes, and the change in the level of one of them may lead to a change in the concentration of the other [61]. 

There was a positive correlation between BMI and serum cysteine level. Other groups observed this phenomenon as well, indicating that the plasma cysteine level was a single, strong determinant of BMI [62]. The levels of aspartate, ethanolamine, glycine, and serine negatively correlated with BMI. In the case of glycine, this type of correlation was documented by Badoud et al. [23], but no correlation with the BMI was published so far, concerning the other amino acids. The WHR can reflect insulin resistance [63] and positively correlates with the concentration of BCAA: isoleucine, leucine, and valine. These amino acid levels are markers of insulin resistance according to the data published in the literature [64]. The level of BCAA was related to hyperlipidemia and obesity-related insulin resistance as well [64], a phenomenon also present in our datasets. We observed a positive correlation of isoleucine and leucine levels with the level of triglycerides and the negative correlation of isoleucine, leucine, and valine with the HDL. These data are in accordance with the clinical laboratory results of patients with T2D, where high triglyceride and LDL levels along with low HDL levels were reported [65]. The positive correlation of triglyceride levels with alanine and cysteine and the negative correlation with glycine levels were also confirmed in our study. 

The cysteine concentration correlated positively, while the threonine concentration negatively with CRP levels. It was shown that CRP can be a predictor of cardiovascular risk and may participate actively in atherogenesis [66], and its association with cysteine concentration was found by other groups as well [67]. Because cysteine is required for glutathione synthesis and it was shown that glutathione and CRP have synergistic effects on the progression of liver cirrhosis [68], we can speculate on the possible link between the cysteine and CRP levels and that they might predict liver-related complications. Regarding threonine, no significant correlation with CRP has been documented so far. 

The level of fibrinogen, which is higher in diabetes [69], negatively correlated with the levels of histidine and threonine. We observed a positive correlation between the concentration of homocysteine and citrulline and a negative correlation between the ApoA1 and tyrosine, HDL, and phenylalanine levels. The parameters reflecting kidney functions, such as albumin/creatinine ratio (ACR) along with the GFR, negatively correlated with serum ethanolamine levels. The association of some of these parameters such as HDL and phenylalanine [67] was demonstrated by other groups but, searching the scientific literature, we could not find similar correlations between fibrinogen, homocysteine, and ApoA1 levels and the concentration of amino acids in diabetes or obesity. At the moment, we can only speculate on their importance in these two pathological conditions without being able to give exact information. The concentration of glycated hemoglobin and isoleucine correlated; this correlation can be explained by the increased BCAA levels in diabetic patients, where usually the level of HbA1C is higher [70]. Regarding the GFR, along with the ethanolamine levels, the negative correlation with glycine levels could also be detected. We could notice the association of both metabolites with BMI; their levels negatively correlated with it. It was shown that in obesity, the GFR increases with BMI, and when bariatric surgery is applied to reduce the BMI, the GFR is also reduced [71]. 

#### 2.1.4. Network Analysis

Network analysis is a widely used method to examine proteomics, transcriptomics, and metabolomics datasets and it can provide extensive information on the studied system and help to better understand the pathophysiological events laying behind the examined conditions [72,73,74]. To be able to find the information behind the changes in the amino acid levels and to better understand the impact of the observed differences, we have carried out a network analysis. 

The metabolic pathways of each amino acid changes in a statistically significant manner between the control and either obese or T2D groups were considered, and the enzymes involved in the metabolism of these amino acids were retrieved. The transporters having a role in transmembrane transport of the respective amino acids were also retrieved, and a list of proteins was generated containing both the metabolic enzymes and the transporters modulating the levels of the selected amino acids. The first shell interactors of the enzymes and transporters were acquired using STRING DB [75], and their protein–protein interaction networks were generated. To obtain functional information, the enriched GO terms were examined using the ClueGO v2.5.7 (Figure S1) and the gene interaction data of CluePedia v.1.5.7. (Figure S2).

Considerable overlap between the obese and T2D networks could be observed. After excluding the overlapping GO terms, the functions characteristic of T2D were related to the metabolism of glutamine and glycine, cellular respiration, and NO synthesis, while the response to fatty acid and sulfur compound metabolism were specific to obesity (Figure S1). 

Considering the gene interaction networks, several distinct clusters were identified. The cluster of amino acid metabolic enzymes (1), the cluster of proteins having a role in retrograde transport including sorting nexins and VPS proteins (2), the cluster containing choline and ethanolamine kinases (3), and the cluster of iron-binding proteins (4) could be observed in both networks (Figure S2). 

We do not have exact information on the role of the retromer complex in diabetes and obesity, but some findings suggest the involvement of the retrograde transport from endosomes to Golgi in the recycling of GLUT4 [76,77]. In a mouse model of diabetes, decreased Vps35 levels were found [78], and a single nucleotide polymorphism at the Vps26 locus was identified in T2D [79]. In addition, the downregulation of sortilin expression was described in obese humans [80,81]. All these data suggest the importance of the recycling of GLUT4 from endosomes by sortilin- and retromer-mediated processes in the development of insulin resistance. 

The role of choline and ethanolamine kinases in obesity and diabetes might be related to the altered phospholipid metabolism observed in insulin resistance [82]. These data give further evidence to the clinical observation that the patients with obesity recruited into this study already had insulin resistance.

The proteins FDX1, 2, FXN, ISCU, and LYRM4, with a role in the iron–sulfur cluster assembly, formed a distinct cluster in the obese network, while the smaller cluster of FXN, ISCU, and LYRM4 was linked to the NOS-containing cluster in the T2D network (Figures S2 and S3). The role of the iron–sulfur clusters and their assembly was already linked to altered energy generation observed in diabetes and obesity [83,84].

Clusters specific to either T2D or obesity were also observed. The cluster of solute carrier family members was present only in obese networks, while the NOS-containing cluster and the cluster of beta-oxidation enzymes (ACADVL, ACAA2, HADHB) were only in T2D networks. 

Some of the solute carriers such as SLC7A10, SLC7A11, and SLC7A9 were previously demonstrated to have importance in obesity [85,86,87]. Their altered expression might be responsible for the differences in the serum amino acid levels observed.

**Figure 3:**
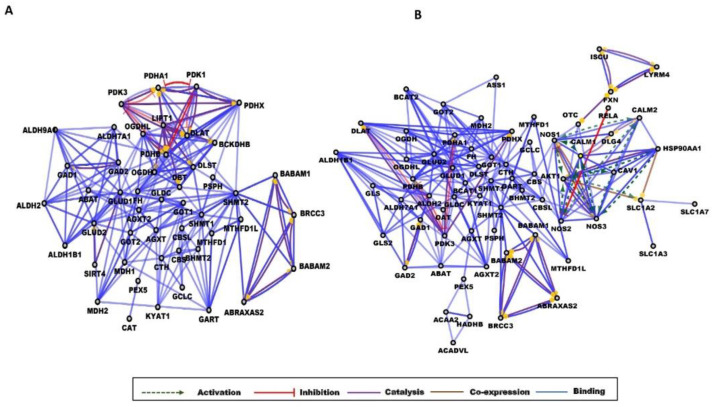
Gene interaction networks in obesity and diabetes. Partial network view for (**A**) obese and (**B**) T2D networks. The circles represent a gene/protein and the lines indicate interactions. The lines with an arrow represent activation, blocking lines represent inhibition, and simple lines represent protein–protein interaction. Line color indicates the type of interaction: green color refers to activation, red color to inhibition, blue color to binding, brown color to co-expression, and purple color to catalysis. On all panels, the proteins are labeled with their gene name. The full network images of these networks are presented in Figure S2.

A common complication of T2D is endothelial dysfunction having a role in the vascular complications related to T2D [88]. Inflammation and the dysfunction of NO synthesis were already linked to endothelial dysfunction, cardiovascular complications, and neuropathy in patients with T2D [89,90,91]. In diabetic rat models, some groups reported an enhanced expression of endothelial NO synthase (NOS3) [92], and some described its downregulation [90,93], indicating the existence of a complex regulatory mechanism. According to our data, a striking difference between the obese and T2D networks was the presence of a cluster containing NO synthases and the enzymes associated with them. In this highly interconnected cluster, we could detect multiple mutual activations between the proteins involved. Calmodulin (CALM1,2), shown earlier to induce diabetes in rodent models [94,95] activated NOS enzymes, and the NOS1, 2, and 3 activated CALM1 and CALM2, respectively (Figure 3). The advanced glycation end products elevated in diabetes can lower the expression of NOS3 and reduce the synthesis of NO [90], but the high glucose level, through the downregulation of caveolin 1 (CAV1), can indirectly decrease the NOS3 expression [93]. AKT1, a kinase activated during the insulin signaling pathway, was activated by NOS 1, 2, and 3 and HSP90AA1 according to our data, and AKT1 activated HSP90AA1, SLC1A2, and caveolin 1 (Figure 3). Some other proteins of this cluster were associated with T2D: HSP90 can be a target for antidiabetic therapies, as its inhibition reversed hyperglycemia [96] and DLG4 was implicated in the regulation of hepatic insulin resistance [97] in rodent models. RELA is a component of the NF-κB, whose activation was linked to the appearance of diabetic nephropathy, a common complication of T2D [98]. It was stated that high glucose levels alone can induce increased NF-κB activity [99], which in turn will lead to inflammation and fibrosis succumbing finally to diabetic nephropathy [98]. 

Regarding the cluster of beta-oxidation enzymes observed in the T2D network, there is scientific evidence that beta-oxidation is impaired in T2D [100]. A study showed that the accumulation of fatty acids, such as long-chain acyl-CoAs, can lead to alterations in insulin signaling [101], which might explain the involvement of beta-oxidation enzymes in T2D.

Besides the clusters, we could observe enzymes specific for either obese or T2D networks at the level of individual proteins. DBT, MDH1, and PDK1 enzymes related to energy production, aldehyde dehydrogenase ALDH9, branched-chain ketoacid dehydrogenase BCKDBHB, catalase, ferredoxin (FDX)1 and 2, lypoil transferase 1 (LPT1), sirtuin 4, and sorting nexin 5 were present only in the obese network, while the BCAA-degrading enzymes BCAT1 and BCAT2, glutaminases GLS and GLS2, argininosuccinate synthase (ASS1), and ornithine aminotransferase (OAT) were unique to the T2D network.

To obtain more information on the key proteins of the networks, the top 20 hub proteins were listed and visualized using the cytoHubba application (Figure S3). These data are in agreement with the gene interaction network analyses. Apart from the overlaps observed, the hub proteins having central roles in the obese network were proteins related to energy production (FH, OGDH, OGDHL, MDH1, DBT, DLST, and BCKDHB), while enzymes having a role in amino acid metabolism (BCAT1, BCAT2, GLUD1, GLUD2, GOT2, and GLDC), nucleotide metabolism (GART), and NO synthesis (NOS3) occupied a central place in the T2D network. 

Regarding obesity, in a study carried out by Satapati and coworkers, in the liver of high-fat-diet-fed mice, elevated gluconeogenesis and mitochondrial citric acid cycle anaplerosis were reported [102]. In our study, the metabolic alterations observed in obesity were mainly linked to energy production. The enzymes specific for the obese networks along with the hub proteins that occupy a central role in the network were all associated with the citric acid cycle, giving further evidence to the link of obesity and altered core metabolic processes.

It is well known that the degradation of BCAAs is impaired in T2D; upon insulin resistance, the muscle protein degradation is increased, and the degradation of BCAAs is decreased, leading to higher serum levels of isoleucine, leucine, and valine [13]. Leucine in turn can help the insulin secretion in pancreatic beta-cells via the mTOR pathway [103], improving glycemic control [104]. The central role of branched-chain aminotransferase (BCAT1, 2) enzymes in the T2D network indicates their importance, but according to the network model applied, we do not have information on the direction of changes. However, taking into account the results of the correlation analyses and the data published in the scientific literature, it is very likely that the decreased degradation of BCAA leads to their increased concentration in serum. Glutamine is a long-studied metabolite in diabetes [105] demonstrated to improve insulin resistance [106]. Some studies have aimed at improving insulin sensitivity and diabetes-related metabolic alterations by the supplementation of either leucine or glutamine, but more studies are needed to clarify their exact effects [107,108,109]. We could not find any information on the direct involvement of the urea cycle enzymes in diabetes. The levels of arginine and ornithine were associated with the risk of T2D in Chinese adults [110]. We can only speculate whether the urea cycle enzymes reflect the liver-related complications of T2D and hence have the potential to predict these complications. Of course, further studies are needed to verify this idea.

The results of the network analyses are consistent with the results of the correlation analyses and the data found in the scientific literature indicating distinct features characteristic of obesity and T2D, respectively. 

### 2.2. Examination of Tear Metabolome

Besides serum, the tear is an emerging biofluid with a high potential in non-invasive investigations. We examined tear metabolites, including amino acids from samples of T2D patients, and observed significant differences between tear samples collected from healthy and T2D patients [45].

We carried out the same analyses on the tear as we performed on serum samples. The major difference between the two sample types was that we could not collect tear samples from the controls. In this way, our setup allows only for the comparison of tear metabolites between the obese and T2D groups, dividing this latter group into those having DR, the most common eye-related complication of DR, and those without any signs of DR.

**Figure 4:**
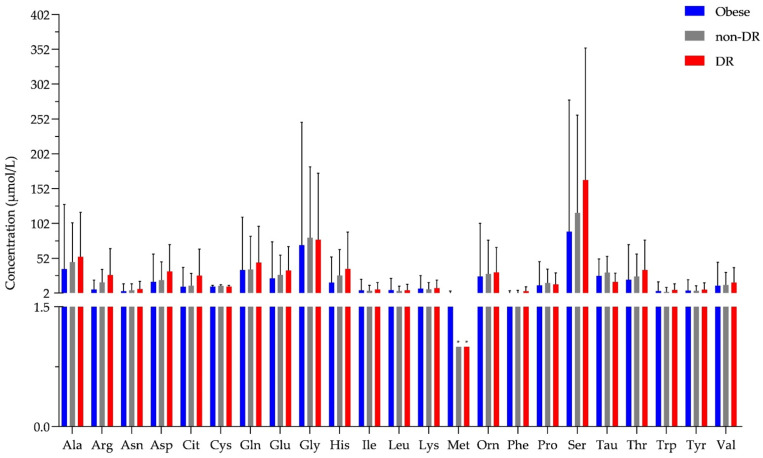
The concentration of amino acids in tear. The y-axis shows the concentration in μmol/L of all 23 amino acids shown on the x-axis. * shows amino acids that were detected but could not be quantified and an arbitrary value of 1 was assigned. Blue bars represent the obese group, the gray bar indicates the T2D group without diabetic retinopathy (non-DR), while the red bars represent the T2D group with diabetic retinopathy (DR).

All the previously examined amino acids were identified and quantified in tear samples from patients with obesity and T2D (Figure 4, Table S1). 

Regarding the biogenic amines, 9 out of 10 were identified in tears, but only ethanolamine concentration was high enough to be quantified in most of the samples (Table S1), showing no statistically significant change between the studied groups.

Small molecule tear biomarkers characteristic of DR would have high importance in the routine clinical diagnosis. To identify new potential biomarkers, the concentration of the examined amino acids and biogenic amines characteristic of the examined groups was compared using statistical analysis. The values obtained by the examination of tear samples originating from patients with obesity, diabetes with no signs of DR, or diabetes with non-proliferative DR were examined, but no statistically significant differences could be demonstrated between the studied groups. 

The correlation analysis performed on serum metabolites was carried out on tear metabolites as well, but none of the correlations were statistically significant (Table S3).

Comparing the tear and serum results, we could see higher values in serum in the case of most amino acids, but the level of aspartate in the obese and T2D group and the level of serine in the T2D group was higher in tear. The level of tryptophan and ethanolamine in both the obese and T2D group and of citrulline in the T2D group was in the same range in the two sample types (Table S1). Our data indicate the utility of tear as a body fluid for metabolomics examinations, and the observed changes are very likely due to the inherent difference between the two body fluids. Very different factors control the levels of metabolites in serum and tears, and the differences seen might be the result of the differential regulation. It also should be mentioned that the method applied in our study is sensitive enough for amino acids, but its sensitivity toward the biogenic amines should be further improved to quantify and not only detect their levels.

According to our data, there was no statistically significant difference between the T2D and obese groups, and tear metabolomics could not distinguish between diabetic patients with or without DR. Regarding the serum analyses, the observed differences in case of obese or T2D groups were statistically significant only in comparison to the control group. Tear proteomics previously published by our group could distinguish various stages of DR, and the most reliable and highest changes were observed between the group without DR and the advanced, proliferative stage of DR [27]. In the current patient cohort, none of the volunteers had a proliferative stage of DR, which might explain our results at the level of the examined metabolites.

## 3. Materials and Methods

All the reagents and solvents used during the study were purchased from Sigma (St. Louis, MO, USA) if not indicated otherwise.

### 3.1. Study Subjects and Sample Collection

In total, 85 subjects were recruited for this study, with 26 patients with T2D, 31 individuals with obesity, and 28 healthy volunteers. The study was approved by the Ethics Committee of the University of Debrecen, and all participants provided written informed consent. The groups were age- and sex-matched; the diabetic group’s average age was 54 years, with a “male–to–female” ratio of 1:1; the obese group’s average age was 53 years, with a “male–to–female” ratio of 1:1; and the healthy group’s average age was 55 years, with a “male–to–female” ratio of about 1:1. 

Fasting blood samples were collected from all participants in tubes without anticoagulants and centrifuged to extract the serum. Sera were aliquoted and stored at −70 °C until they were processed. 

Basal tear samples from 40 of the 85 participants (obese: *n* = 19; T2D without DR *n* = 11; T2D with DR: *n* = 10) were collected using a glass capillary [42] and centrifuged, and the supernatant was kept at −70 °C until the examination. 

### 3.2. Sample Processing

To eliminate macromolecules from the serum, 100 μL of serum sample was filtered using a Nanosep 3 kDa spin column (Pall Corp, New York, NY, USA) at 12,800× *g*, 4 °C for 10 min, and the filtered serum was used for the analysis. In the case of tear samples, 3 μL of tear was diluted with Milli-Q (Millipore, Bedford, MA, USA) water to 50 μL, filtered similarly to the serum sample, and then completely dried in a vacuum centrifuge (ThermoScientific, San Jose, CA, USA). 

### 3.3. Amino Acid and Biogenic Amine Analysis

Twenty proteinogenic (His, Asn, Ser, Gln, Arg, Gly, Asp, Glu, Thr, Ala, Pro, Cys, Lys, Tyr, Met, Val, Ile, Leu, Phe, and Trp), three non-proteinogenic amino acids (Tau, Cit, and Orn), and ten biogenic amines (histamine, ethanolamine, methylamine, ethylamine, putrescine, serotonin, cadaverine, tyramine, tryptamine, and phenethylamine) were analyzed as described by Guba et. al. [111] after the sample was derivatized with AccQ-Tag Ultra derivatization kit according to the manufacturer’s protocol (Waters, Milford, MA, USA). 

Briefly, 60 μL of AccQ-Tag Ultra borate buffer and 20 μL of AccQ-Tag derivatization reagent were mixed with 20 μL of analyte-containing solution used for calibration. A 10-point calibration curve was prepared containing 0.25, 0.5, 1.0, 2.5, 5.0, 7.5, 10.0, 15.0, 20.0, and 30.0 micromol/L of analytes, respectively and used to determine the concentration of each analyte. 

For the derivatization of serum samples, 10 μL of filtered serum was mixed with 70 μL of AccQ-Tag Ultra borate buffer and 20 μL of AccQ-Tag derivatization reagent. For the derivatization of the tear samples, the dried samples were resuspended in 80 μL AccQ-Tag Ultra borate buffer and 20 μL of AccQ-Tag derivatizing reagent was added.

After adding the derivatizing reagent into the glass vials, all samples and calibration standards were incubated at +55 °C degrees for 10 min and analyzed on Acquity H-class UPLC system (Waters, USA) coupled to 5500 QTRAP (Sciex, Framingham, MA, USA) mass spectrometer.

One microliter of the sample was injected, and two technical replicates were recorded. The chromatographic separation was performed on a column (AccQ-Tag^TM^ ULTRA C18 1.7 µm, 2.1 × 100 mm) using an in-house developed 11-min gradient [111]. Double detection was performed; the derivatized analytes were detected at 260 nm wavelength by a PDA detector and by the mass spectrometer working in MRM mode, respectively [111]. 

### 3.4. Data Analysis

The analytes were identified based on their retention time and verified using the MRM transitions. Where it was possible, the UPLC data were used for quantification with the Empower.v3 (Waters, USA) software. In the case of analytes with lower concentrations than the detection limit of the UPLC, the mass spectrometry data exported to the Skyline (v.20.2, www.maccosslab.org, downloaded on 21 January 2022) were used. The area under the curve (AUC) of each analyte was extracted and used for further examinations.

For the statistical analysis of the data, we applied a one-way ANOVA analysis to test the significantly different analyte quantities between the investigated groups. After running post-hoc Tukey’s tests to determine the *p*-values of group differences, we retained the significant results with an FDR < 0.05 criteria.

For correlation analysis, we applied non-parametric Spearman correlation tests to study the association between the analytes and the other clinical data of the investigated population. For considering the Type-I error, we applied FDR corrections for the same data sources and reported only those associations in which FDR-corrected *p*-values were less than 0.05. 

### 3.5. Network Analysis

To create the interaction network of enzymes related to the selected amino acids and biogenic amine, we first obtained all corresponding degradation and, where applicable, biosynthesis pathway identifiers from MetCyc (MetaCyc.org) [112] as part of the BioCyc (BioCyc.org) database collection. We retrieved all available enzyme annotation of the pathways of interest via MetaCyc’s application programming interface (API) with the brendaDb R package (v1.6.0) [113]. We used R statistical software (v4.0.3) for table operations and reorganization of the downloaded data [114]. The enzyme dataset was complemented with the relevant amino acid transporters based on a comprehensive review article [115]. We queried the STRING database (v11.5) [75] with the updated protein list. The generated protein network at a 0.9 confidence level, with up to 50 among the first shell of interactors, included physical and functional associations, documented only in experiments and databases as active interaction sources. We imported the T2D and obesity-related amino acid metabolic enzymes and transporters and their first shell of interactors into the Cytoscape’s v3.9.0 [116] app ClueGo v2.5.8 [117] for pathway analysis. ClueGo parameters were set to *p*-value ≤ 0.05, and the threshold for CluePedia gene visualization was set to 1000. All proteins were searched using the GO_biological pathways database. Next, CluePedia v1.5.8 [118] analysis was performed. We processed the data using CluePedia and examined them for the five interaction types retrieved from the String-DB v11.5. The interactions examined were activation, inhibition, catalysis, binding, and co-expression. The Cluepedia network was further investigated using Cytohubba v0.1 [119] to determine the top hub proteins of the network. Based on the Matthews correlation coefficient scoring, Cytohubba creates the networks for the top hub proteins. These top hub proteins were further explored using Cluepedia to determine the interactions between them. The Style menu was used to visualize the network of top-hub proteins. We labeled the amino acid modifying enzymes and transporters with a circle and the first shell of interactors with a triangle.

## 4. Conclusions

In our metabolomics study, we intended to look behind the statistically significant metabolite changes and correlation analysis data, and we were eager to know if new pathways and functions specific to the disease conditions could be identified using network models (Figure 3 and Figures S2 and S3). With our network analyses, we could further highlight the common pathological traits observed in both obese and T2D groups, and new pathways could also be found. The problems related to ethanolamine metabolism and the involvement of the retrograde transport via retromer were characteristic of both conditions (Figure S2). Of course, further studies are needed to test the idea, but the retromer-mediated retrograde transport, the ethanolamine metabolism, and consequently the endocannabinoid signaling [59], and phospholipid metabolism [82] can be important targets for future therapies aiming to alleviate insulin resistance. This might be the case in the advanced forms of obesity when insulin resistance has already been developed. However, it would be interesting to see if these pathways and functions appear in early obesity and whether their presence has a predictive function in the appearance of insulin resistance.

Using the applied network model, we could monitor the differences between obese and T2D groups. In obesity, the alterations related to energy generation, while in T2D the deep involvement of the NO synthesis and its relation to insulin signaling and inflammation were the most prominent functions. The implication of enzymes with a role in amino acid metabolism, especially the metabolism of BCAA, glutamine, the urea cycle, and beta-oxidation, were also characteristic of T2D.

With the application of the network model, new functions previously hidden in the data acquired by the examination of the concentration changes of the amino acid and biogenic amines emerged. This phenomenon emphasizes the importance of the application of complex data analysis procedures to better understand pathological conditions.

It is also important to highlight that with our analytical workflow we could obtain extensive information about the metabolic statuses of the patients. Some of these results were already available, arising from various, often hard-to-implement experiments. The current metabolomics analysis along with the network model applied could provide these data in one study.

We are aware that further studies are needed to test the emerged ideas and to acquire more information on the complex metabolic dysregulation during obesity leading to insulin resistance and T2D. One of the main limitations of our study is that more donors need to be recruited to be able to involve patient stratification. Our data emphasize the importance of control group in study design, as no difference between the obese and T2D groups could be detected in the examined sample types. This indicates another limitation of the current study, namely that the patients with obesity recruited to the study already had insulin resistance, as was demonstrated by the metabolomics results. In this way, we could obtain information on the difference between advanced obesity and T2D but not on the metabolic changes leading to insulin resistance. More studies are needed to examine the early changes, with potential predictive value, leading to insulin resistance, and the results should be validated on independent cohorts. It is also important to mention that the current method worked well for amino acids but was not sensitive enough for biogenic amines. Three biogenic amines could be detected and two of them could be quantified in serum; and nine could be detected, but none of them could be quantified, in tears. Although we could not detect statistically significant differences between the groups in tears, the levels of some amino acids were in the same range or even higher in tears compared to serum, further highlighting the utility of tear for metabolomics analyses. The method applied in our study is very likely not robust enough for tear metabolomics analyses, as the network analysis could be conducted based on the serum results only. However, it cannot be excluded that the results of tear analyses are distorted by the relatively low number of tear samples available, and by recruiting more volunteers for tear analysis, results useful for network analysis could be generated. 

Despite the above-mentioned limitations, our results can provide information that can be used as potential targets for mechanistic studies aiming at developing future therapies for insulin resistance observed in advanced obesity and T2D.

## Figures and Tables

**Table 1:** Correlation analysis of the clinical and demographical parameters with the examined serum amino acid and biogenic amine concentrations. The table shows the statistically significant correlations. The correlation coefficient, *p*-value, and FDR-corrected q value are shown in each correlation; lines in italics indicate a negative correlation.

Clinical Parameter	Serum Analyte	Correlation Coefficient (rho)	*p* Value	FDR-Corrected q Value
ACR	Eth	−0.41	0.00213	0.04408
ApoAI	Tyr	−0.34	0.00169	0.04027
BMI	Eth	−0.45	0.00006	0.00582
BMI	Gly	−0.38	0.00077	0.02288
BMI	Asp	−0.36	0.00166	0.04027
BMI	Ser	−0.34	0.00251	0.04718
BMI	Cys	0.64	0.00000	0.00000
C peptide	Gly	−0.43	0.00007	0.00593
C peptide	Ser	−0.37	0.00067	0.02093
CRP	Thr	−0.33	0.00225	0.04514
CRP	Cys	0.36	0.00085	0.02392
Fibrinogen	His	−0.40	0.00029	0.01289
Fibrinogen	Thr	−0.37	0.00092	0.02479
GFR	Eth	−0.42	0.00048	0.01831
GFR	Gly	−0.38	0.00181	0.04134
HbA1C	Ile	0.35	0.00147	0.03804
Hcys	Cit	0.33	0.00237	0.04607
HDL	Leu	−0.50	0.00000	0.00036
HDL	Ile	−0.49	0.00000	0.00043
HDL	Val	−0.40	0.00016	0.00920
HDL	Phe	−0.37	0.00052	0.01831
HOMA	Gly	−0.50	0.00012	0.00759
Insulin	Gly	−0.55	0.00000	0.00003
Insulin	Ser	−0.39	0.00028	0.01289
Triglyceride	Gly	−0.34	0.00186	0.04134
Triglyceride	Ala	0.34	0.00194	0.04158
Triglyceride	Cys	0.37	0.00061	0.01983
Triglyceride	Leu	0.37	Triglyceride	0.01831
Triglyceride	Ile	0.41	0.00012	0.00759
WHR	Val	0.45	0.00053	0.01831
WHR	Leu	0.48	0.00024	0.01226
WHR	Ile	0.51	0.00008	0.00605

The abbreviations are as follows: amino acids are indicated using their three-letter codes, ACR: albumin-creatinine ratio, ApoA1: apolipoprotein A1, BMI: body mass index, CRP: C-reactive protein, GFR: glomerular filtration rate, HbA1C: glycated hemoglobin, Hcys: homocysteine, HDL: high-density lipoprotein, HOMA: homeostatic model assessment of insulin resistance, LDL: low-density lipoprotein, WHR: waist-to-hip circumference ratio.

## Data Availability

The data presented in this study are available on request from the corresponding author.

## Supplementary Materials

The following supporting information can be downloaded at: https://www.mdpi.com/article/10.3390/ijms23094534/s1.
