# Supplementary figures and images for: Metabolomic Analysis of Serum and Tear Samples from Patients with Obesity and Type 2 Diabetes Mellitus
Source: Int J Mol Sci. 2022 Apr 20;23(9):4534. doi: 10.3390/ijms23094534 (PMC9105607; doi:10.3390/ijms23094534)

A

% genes per group

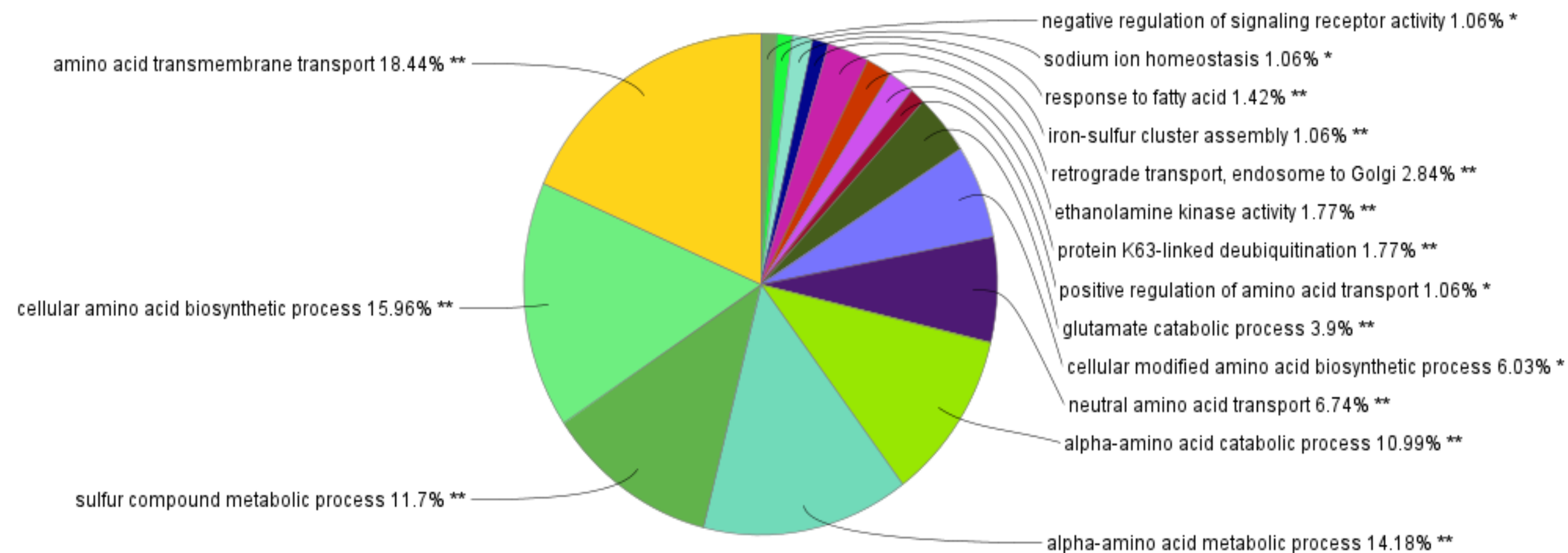

B

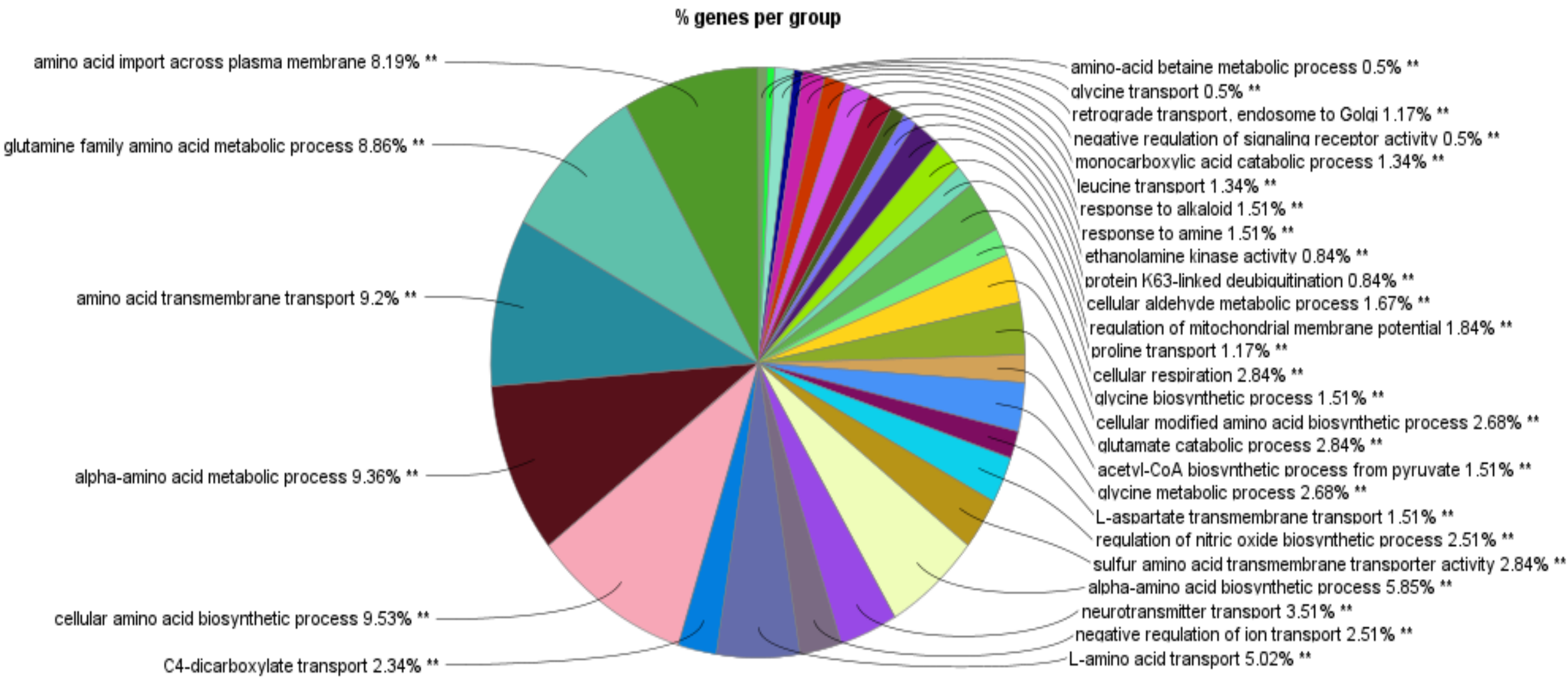

Supplement: Supplementary file 1 [file ijms-23-04534-s001.zip › Figure S1.pdf]

A

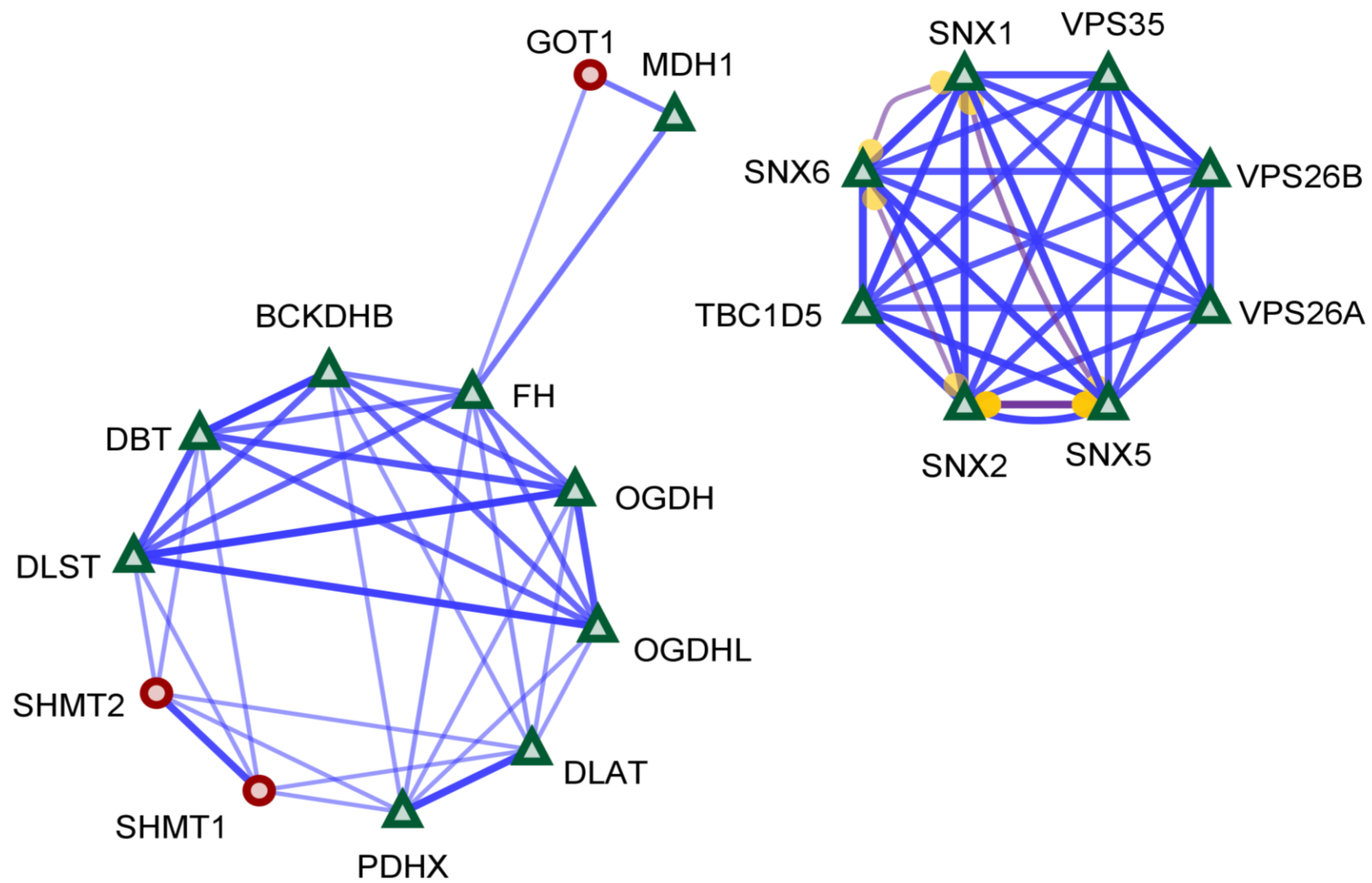

B

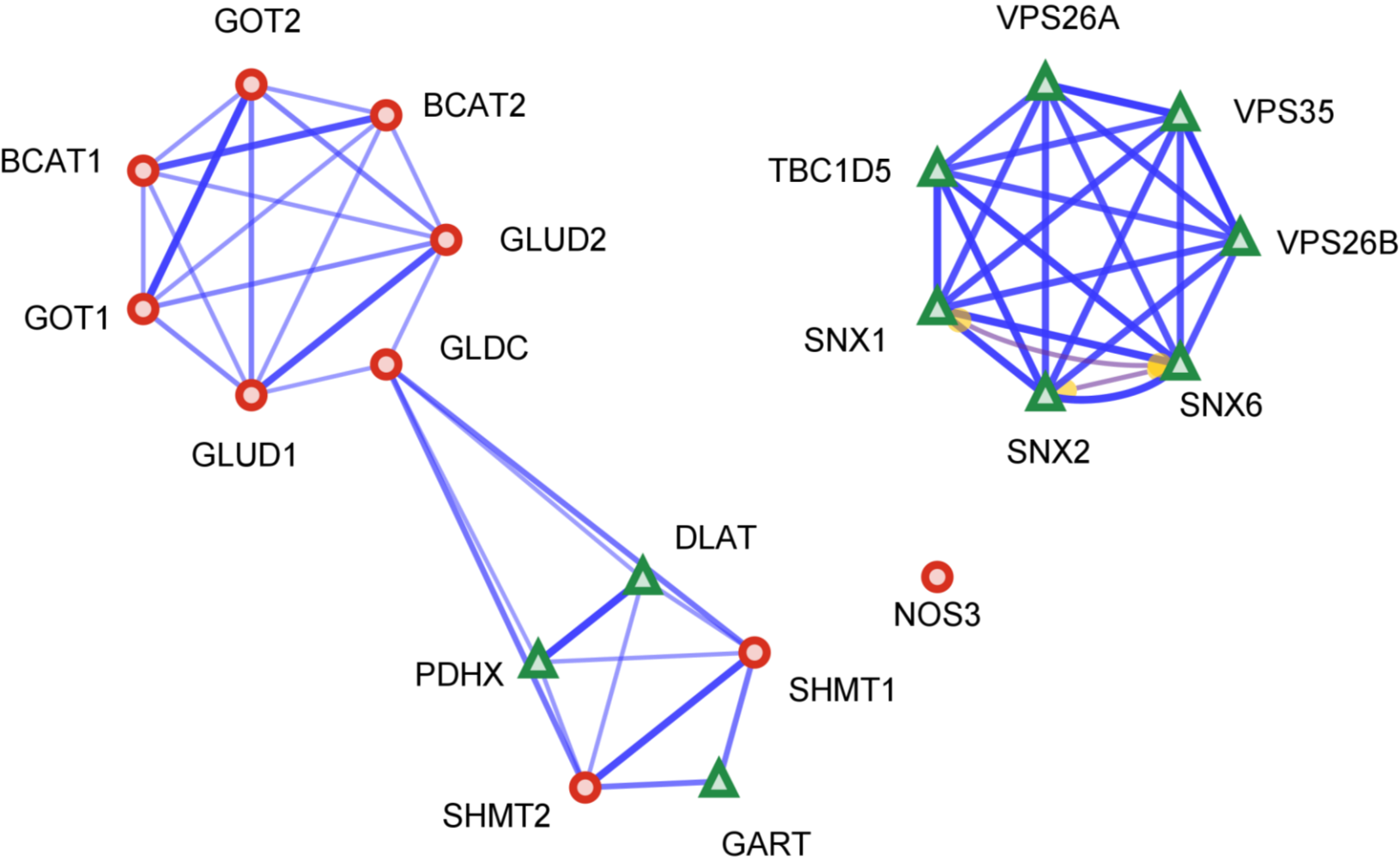

Supplement: Supplementary file 1 [file ijms-23-04534-s001.zip › Figure S3.pdf]

A

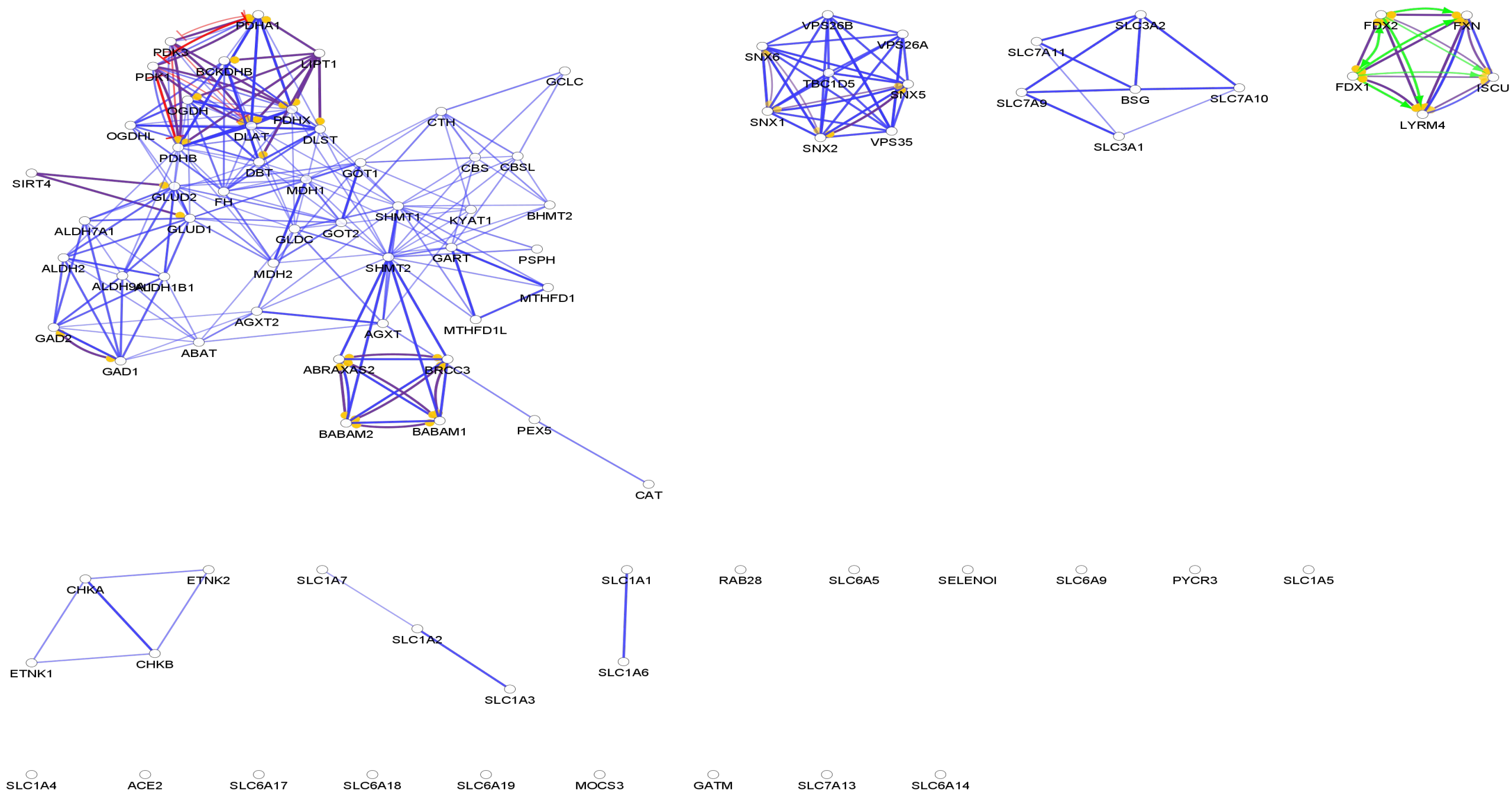

**B**

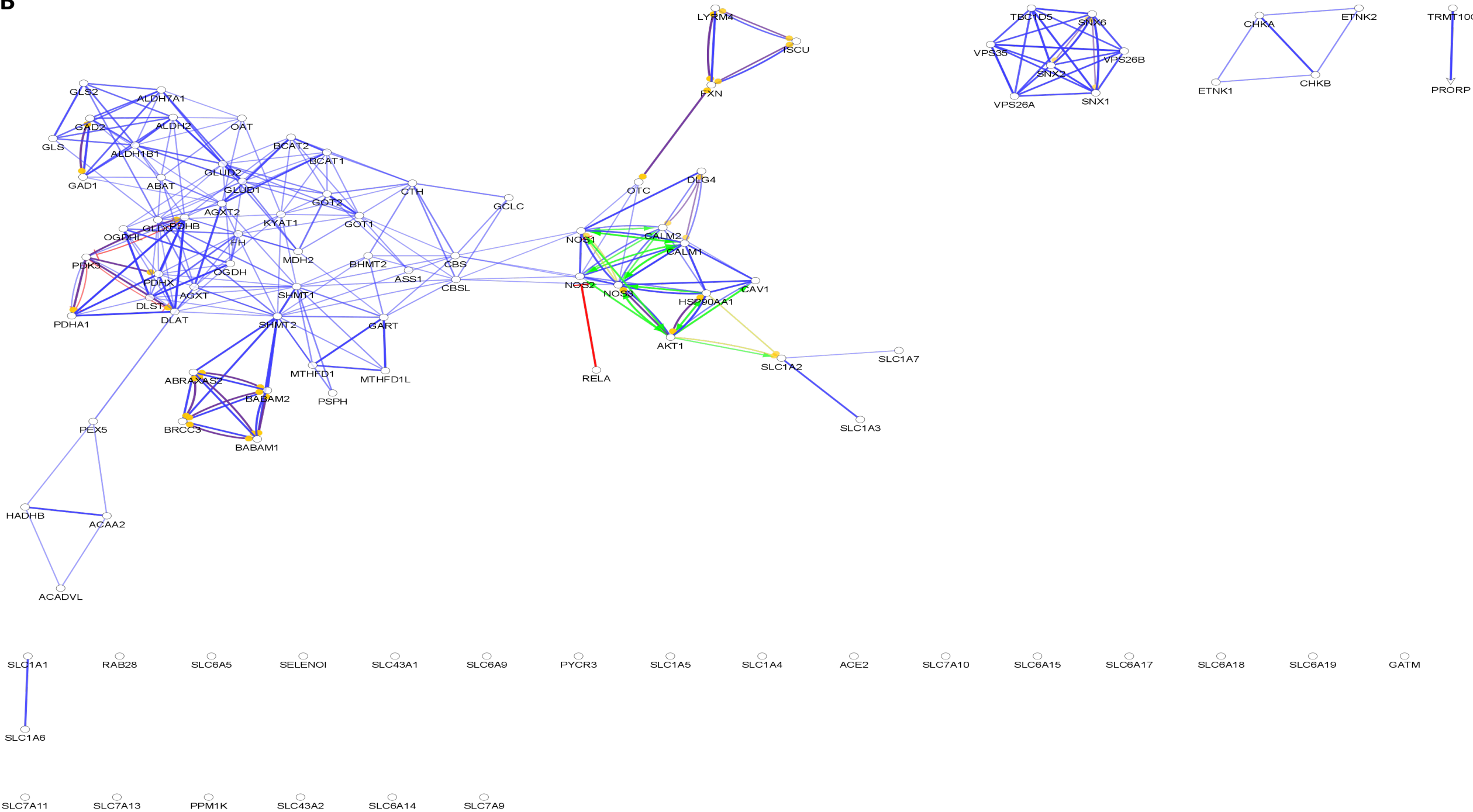

Supplement: Supplementary file 1 [file ijms-23-04534-s001.zip › Figure S2.pdf]
